# Evaluation of nanobody-based biologics targeting purinergic checkpoints in tumor models *in vivo*

**DOI:** 10.3389/fimmu.2022.1012534

**Published:** 2022-10-19

**Authors:** Mélanie Demeules, Allan Scarpitta, Romain Hardet, Henri Gondé, Catalina Abad, Marine Blandin, Stephan Menzel, Yinghui Duan, Björn Rissiek, Tim Magnus, Anna Marei Mann, Friedrich Koch-Nolte, Sahil Adriouch

**Affiliations:** ^1^ University of Rouen, INSERM, U1234, Pathophysiology Autoimmunity and Immunotherapy (PANTHER), Normandie Univ, Rouen, France; ^2^ Institute of Immunology, University Medical Center Hamburg-Eppendorf, Hamburg, Germany; ^3^ Core Facility Nanobodies, University of Bonn, Bonn, Germany; ^4^ Mildred Scheel Cancer Career Center HaTriCS4, University Medical Center Hamburg-Eppendorf, Hamburg, Germany; ^5^ Department of Neurology, University Medical Center Hamburg-Eppendorf, Hamburg, Germany

**Keywords:** P2X7, purinergic signaling, tumor microenvironment (TME), anti-tumor immune responses, nanobody, nanobody-based biologics, AAV vectors

## Abstract

Adenosine triphosphate (ATP) represents a danger signal that accumulates in injured tissues, in inflammatory sites, and in the tumor microenvironment. ATP promotes tumor growth but also anti-tumor immune responses notably *via* the P2X7 receptor. ATP can also be catabolized by CD39 and CD73 ecto-enzymes into immunosuppressive adenosine. P2X7, CD39 and CD73 have attracted much interest in cancer as targets offering the potential to unleash anti-tumor immune responses. These membrane proteins represent novel purinergic checkpoints that can be targeted by small drugs or biologics. Here, we investigated nanobody-based biologics targeting mainly P2X7, but also CD73, alone or in combination therapies. Blocking P2X7 inhibited tumor growth and improved survival of mice in cancer models that express P2X7. P2X7-potentiation by a nanobody-based biologic was not effective alone to control tumor growth but enhanced tumor control and immune responses when used in combination with oxaliplatin chemotherapy. We also evaluated a bi-specific nanobody-based biologic that targets PD-L1 and CD73. This novel nanobody-based biologic exerted a potent anti-tumor effect, promoting tumor rejection and improving survival of mice in two tumor models. Hence, this study highlights the importance of purinergic checkpoints in tumor control and open new avenues for nanobody-based biologics that may be further exploited in the treatment of cancer.

## Introduction

Adenosine triphosphate (ATP) release into the extracellular space (eATP) represents a well-known danger signal acting through two main families of plasma membrane receptors: G protein-coupled receptors, named P2Y receptors, and ATP-gated ion channels, termed P2X receptors (1). Among the latter family, P2X7 forms a homotrimeric receptor that has attracted much interest in the fields of inflammation and cancer. P2X7, a non-selective ligand-gated cation channel, is expressed at the cell surface of various leukocytes, in particular monocytes, macrophages and regulatory T cells (2–5). P2X7 is known to be central in inflammation for its ability to activate the NLRP3 inflammasome and trigger IL-1β and IL-18 release (3, 6–9). Prolonged activation of P2X7 leads to the opening of a membrane pore allowing the entry of large molecules of up to 900 Da. Whether this membrane permeabilization is due to dilatation of the P2X7 channel itself or to the activation of non-selective pores like pannexin-1, gasdermin D or anoctamin 6, may depend on the cellular context, the lipid composition of the membrane, and on the level of expression of these proteins (10–12). Whatever the exact molecular mechanism that leads to pore formation, P2X7 can induce a major perturbation of intracellular ion balance and thereby modify cellular activities, cellular function, and cell fate. In the context of cancer, P2X7 has been assigned various and contrasting roles as a driver of cancer cell growth (13) and metastatic dissemination, or as a promoter of immune mediated tumor eradication (14, 15). High concentrations of eATP, released in the vicinity of stressed or damaged cells during inflammation, but also within the tumor site, represent a “danger signal” than can influence the activity and function of immune cells. P2X7 is also found at the surface of numerous tumor cell types an as been proposed to confer a selective advantage to tumor cells through their tonic stimulation, leading to higher concentration of mitochondrial calcium, fueling growth and invasiveness (16, 17). eATP plays a complex role within the tumor microenvironment (TME) depending on multiple factors such as its concentration, the abundance of ecto-ATPases, the expression level of P2X7, and the nature of the P2X7 variant expressed by immune and tumor cells (18, 19).

On T lymphocytes, P2X7 induces the shedding of CD62L and CD27 by metalloproteases (5, 20–23), and controls the differentiation, proliferation and survival of T cells and of tissue resident memory T cells (24, 25). Also, FoxP3^+^ regulatory T cells (Tregs) are known to express P2X7 at high levels, and its activation controls their phenotype, their suppressive functions, as well as their survival (4, 5). Taken together, activation of P2X7 on myeloid and lymphoid cells converge to promote and amplify inflammation as well as the emergence of an adaptive T cell response (3, 6, 26).

In the TME, P2X7 blockade induces modification in the expression of ecto-enzymes involved in the control of the purinergic signaling cascade, including modifications in the expression of the 5’-ecto-nucleotidase CD73 that control accumulation of immunosuppressive adenosine (27). This strongly suggests that P2X7 expression and function may control the entire eATP/adenosine balance in the TME and may exert a broad impact on tumor proliferation and dissemination. Yet, other studies also suggested a key role of P2X7 in anti-tumor immunity and have linked the release of its ligand, ATP, to the occurrence of immunogenic cell death and to stimulation of anti-tumor immune responses (28, 29). Hence, P2X7 either on its own or together with other key players of the purinergic signaling cascade such as CD39 and CD73, play an important role in tumor immunogenicity at least during the early phase of cancer development (29–31). Antagonistic antibodies targeting CD39 that are in clinical development exert their therapeutic effect partly in relation with P2X7 (32, 33). CD73-blocking antibodies, which also hold promise in clinics, were shown to improve anti-tumor immune responses and to prevent metastasis when used alone or in combination with other therapeutic strategies (34, 35). Indeed, CD73 blockade may decrease accumulation of adenosine, a potent anti-inflammatory molecule that inhibit anti-tumor immune responses and favor tumor growth (36–38). This protumoral effect of adenosine rely on different mechanisms, such as the expression of the inhibitory A2a adenosine receptors by T cells limiting their activation and expansion in the adenosine riche TME (36, 37). These discoveries has led to preclinical and clinical validations that have better delineate the factors that contribute to improve anti-tumor immune responses in therapeutic strategies aiming to inhibit adenosine signaling in the tumor context (39–42).

Taking together, this suggest that targeting P2X7 and/or multiple key purinergic players in the TME, may be beneficial for the treatment of tumors. Here we used an AAVnano methodological approach that we previously described (43, 44) to evaluate the anti-tumor effects of P2X7 blocking, or conversely P2X7 potentiation, in different tumor models *in vivo*. Further, we developed novel nanobody-based biologics targeting other key immune and/or purinergic checkpoints of the TME and evaluated their efficacy either alone, or in combination with a P2X7-potentiating nanobody-based biologic. Interestingly, we demonstrated that a bi-specific construct targeting PD-L1 and CD73 exerts a potent anti-tumor effect, promoting tumor rejection and improving mice survival in two tumor models. Hence, this study highlight the importance of the purinergic signaling in the tumor context and suggests that key purinergic players represent attractive novel anti-tumor checkpoints that may be further exploited in future treatments.

## Material and methods

### Mice, reagents, antibodies

C57BL/6JRj wild-type mice obtained from Janvier Labs were used for all experiments. Mice were housed in a specific pathogen-free facility and were aged of 8 weeks at the beginning of experiments. All animal experimental protocols were approved by the French Ministry of Education and Research, after consultation of the ethical committee (n° APAFIS#27682). Adenosine 5’-tri-phosphate disodium salt was purchased from Sigma Aldrich (A2383). Red blood cell (RBC) lysis/fixation Solution, True-Nuclear Transcription factor buffer set, and antibodies to CD45 (clone 30-F11), CD4 (RM’-5), CD8 (53-6.7), CD25 (PC-61), CD19 (1D3/CD19), FoxP3 (MF-14), CD27 (LG.3A10), CD62L (MEL-14), P2X7R (1F11), CD39 (Duha59), CD73 (CXCR3-176), TIGIT (1G9), TIM3 (RMT3-23), CD49b (DX5), NK1.1 (PK136), CX3CR1 (SAO11F11), PD-1 (29F1A12), CD11c (N418), CD11b (M1170), CD44 (IM7), XCR1 (ZET), P2X7 (1F11) and purified CD16/D32 (TruStain FcX) were obtained from Biolegend or Sony Biotechnology.

### Cell cultures

Mouse B16F10 melanoma (ATCC CRL-6375), mouse Lewis Lung Carcinoma (LLC, ATCC CRL-1642) and mouse thymoma (EG7, ATCC CRL-2113) cell lines were maintained in culture using standard procedures and were regularly tested for the absence of mycoplasma contamination. B16F10 and LLC were grown in DMEM glutamax medium, FBS 10%, penicillin (100 U/ml) streptomycin (100 mg/ml) and pyruvate 1 mM, (all purchased from ThermoFisher Scientific, Gibco). EG7 were grown in RPMI medium, FBS 10%, penicillin (100 U/ml) streptomycin (100 mg/ml) and pyruvate 1 mM, (all purchased from ThermoFisher Scientific, Gibco).

### Flow cytometry analyses

For evaluation of P2X7 expression tumor cells were collected, and single-cell suspensions were prepared and washed using standard procedures. Cells were stained with fluorochrome-conjugated anti-P2X7 (clone 1F11) or related isotype controls.

For evaluation of P2X7-dependent shedding of CD27 and CD62L upon ex vivo exposure to ATP, blood samples were collected, washed, resuspended into PBS (without Ca^2+^ and Mg^2+^) and divided into 3 tubes. Cells were then treated with 30 µM ATP, 150 µM ATP or left untreated. After incubation for 15 min at 37°C, cells were washed in cold D-PBS containing 10% FBS and stained on ice with fluorochrome-conjugated antibodies before one-step fixation and RBC lysis (using RBC Lysis/Fixation Solution, Sony Biotechnology). The percentages of cells co-expressing CD27 and CD62L were then evaluated by flow cytometry.

For the evaluation of the cells infiltrating the tumor, the B16F10 tumor was excised, and the cell suspension was filtered through a 100 µM nylon filter (cell strainer, BD Biosciences). Single cell suspensions were prepared and washed using standard procedures. Cells were stained with fluorochrome-conjugated antibodies before one-step fixation and RBC lysis. Cells were then analyzed by flow cytometry.

Flow cytometry data acquisitions were performed using an LSRFortessa or a FACSCanto-I (BD Biosciences) apparatus and subsequent analyses were performed using FlowJo software (v10.8.1,Tree Star, Ashland, OR).

### Nanobody-based biologics, production of AAV vectors, and muscle transduction

All nanobody-based biologics were based on nanobodies generated and selected as described before (9, 45). The construct 14D5-dimHLE used to potentiate P2X7 function was based on a nanobody dimer (“dim” format) fused to the Alb8 anti-albumin nanobody (to confer half-life extension “HLE”). This construct contains the coding sequences for to two 14D5 nanobodies, fused together using a 35-GS linker (GGGGS)x7, and the coding sequence for an anti-albumin nanobody Alb8 fused *via* a 9-GS linker (GGGGSGGGS) (9, 45). Similarly, PD-L1-dimHLE, CD73-dimHLE and CD73/PD-L1-dimHLE were designed as monospecific or bi-specific nanobody-based biologics based on the same strategy with the published sequence of an anti-PD-L1 blocking nanobody (clone B3) (46) or the sequence of a CD73-blocking nanobody (clone SB121). The CD73-specific nanobody SB121 was generated from an immunized alpaca and reformatted into a dimHLE using established protocols described earlier (47–49). The construct 13A7-hcAb used to inhibit P2X7 function was designed as a heavy-chain antibody (hcAb) by fusing the sequence corresponding to the nanobody 13A7 to the hinge and Fc regions of a mutated mouse IgG1 antibody carrying the “LSF” mutations (T252L, T254S, T256F) described previously to confer higher affinity to the neonatal Fc receptor (FcRn) and thereby an extended half-life *in vivo* (50, 51). For AAV vectors production, all constructs were cloned into a pFB plasmid under the control of a CBA promoter (for AAV1 constructs encoding 14D5-dimHLE and 13A7-hcAb) or under the related CASI promoter (for AAV8 constructs encoding PD-L1-dimHLE, CD73-dimHLE and CD73/PD-L1-dimHLE). Production, purification, and titration of recombinant AAV1 and AAV8 were performed by Virovek (Hayward, California, USA) using the baculovirus expression system in Sf9 insect cells. For muscle transduction, mice hind legs were shaved under anesthesia and 100 µL diluted AAV were injected into muscles using a total dose of 10^11^ viral genomes (vg) per mouse.

### Experiments in murine models

For muscle transduction, mice hind legs were shaved under anesthesia and 100 µL PBS-diluted AAV were injected into 4 muscle sites (gastrocnemius and/or quadriceps) to reach a total dose of 10^11^ viral genomes (vg) per mouse. 21 days later B16F10 (5x10^5^), LLC (5x10^5^) or EG7 (10^6^) cells were subcutaneously injected into the right flank of each mouse. The animals were randomized, and the operator was blinded to the group of allocation. Tumors were measured using a digital caliper, and their volumes were estimated using the formula V= length × width × [(length + width)/2]. In some experiments, 5mg/kg oxaliplatin was injected seven days after tumor inoculation.

### Statistics

All data are presented as mean ± standard error of the mean (SEM). Statistical comparisons between experimental groups were performed using one-way ANOVA and Tukey’s multiple comparisons *post hoc* tests. Two-Way ANOVA with Dunnett’s multiple comparisons *post hoc* tests was used for statistical analysis of tumor growth curves. Statistical analysis of the survival was performed using Log-Rank tests. All statistical analyses were performed with the GraphPad Prism software (V8, GraphPad, San Diego, Ca, USA). For all, the threshold for statistical significance was set at p<0.05 (*p<0.05, **p<0.01, ***p<0.001 and **** p<0.0001).

## Results

### Injection of AAV vectors coding for nanobody-based biologics that target P2X7 significantly blocks or potentiates P2X7 functions *in vivo*

**Figure 1:**
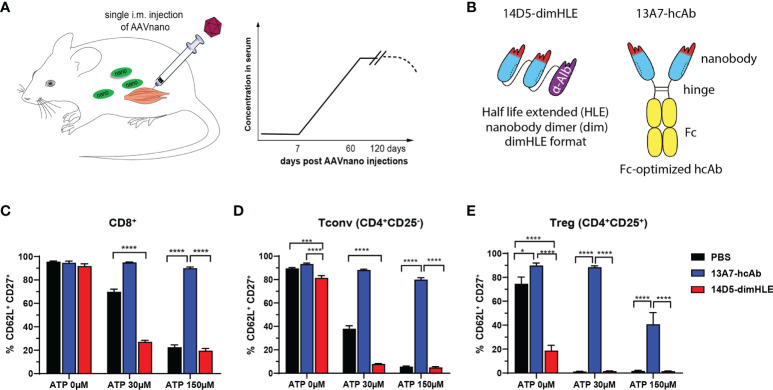
AAVnano methodology used to block or to potentiate P2X7 activity *in vivo* **(A)** AAVnano methodology is based on the single i.m. injection of AAVnano vectors coding for a nanobody-based biologic. This is anticipated to induce long-term and stable *in vivo* production of the designed biologics *in vivo* (43). **(B)** Schemes illustrating the format of the different nanobody-based biologics used in this work. The 14D5-dimHLE is composed of a dimer of the P2X7-potentiating 14D5 nanobody, coupled to a third albumin-specific nanobody (Alb8) conferring extended half-life (HLE). The 13A7-hcAb is composed of an antagonistic nanobody targeting P2X7, coupled to the hinge and the Fc-region of a mouse IgG1, carrying “LSF” mutations (T252L, T254S, T256F) to confer higher affinity to the neonatal Fc receptor (FcRn) involved in extending antibody half-life *in vivo* (50). **(C–E)** The ability of the corresponding AAVnano vectors to induce functional modulation (i.e. inhibition or potentiation) of P2X7 functions *in vivo*, was assessed 20 days after their i.m. injection. For that, blood samples were collected and incubated *in vitro* with 0, 30 or 150 µM ATP. P2X7-dependent shedding of CD62L and CD27 was evaluated at the surface of CD8^+^, CD4^+^CD25^-^ (Tconv), and CD4^+^CD25^+^ (Tregs) lymphocyte subsets, known to express different level of P2X7 and to display increasing sensitivity to ATP. One representative experiment out of at least two is shown with n=7 mice per group. The statistical comparisons between groups were performed using one-way ANOVA. *p<0.05, ***p<0.001, ****p<0.0001.

In this study, we used the AAVnano methodological approach (43, 44), to evaluate the anti-tumor effect of nanobody-based biologics designed to block or to potentiate P2X7 functions. Using this methodology, we previously reported that a single intramuscular (i.m.) injection of the corresponding AAVnano vector elicits production of the modulating nanobody-based biologics over several weeks (43, 44) with a relatively stable pharmacokinetic profile, thereby allowing evaluation of the targeted pathway *in vivo* (**Figure 1A**). We generated AAV vectors coding for a P2X7-antagonistic heavy chain antibody format designated 13A7-hcAb (i.e., nanobody 13A7 fused to the hinge and Fc-region of a mouse IgG1) or a bivalent, half-life extended, P2X7-potentiating nanobody dimer format designated 14D5-dimHLE (i.e. composed of a dimer of nanobody 14D5, fused to a third nanobody specific for albumin) (9) (**Figure 1B**). AAV1 vectors coding for these constructs were injected i.m. in the hind-leg muscles of C57BL/6 mice, 21 days before tumor cell inoculation. First, to evaluate the functionality of the nanobody-based constructs produced *in vivo*, we collected blood samples before tumor inoculation in each experiment, and evaluated P2X7 functionality at the surface of circulating peripheral blood leucocytes. For that, blood samples were incubated with different concentrations of ATP ex vivo, and P2X7-dependent shedding of CD62L and CD27 were monitored on T cells (4, 5, 20, 52, 53). As expected from a P2X7-antagonistic biologic, CD8^+^ T cells (**Figure 1C**), CD4^+^ T cells (**Figure 1D**) and CD4^+^ CD25^+^ regulatory T cells (Tregs) (**Figure 1E**) from mice injected with AAV1 coding for 13A7-hcAb were protected from ATP-induced shedding of CD62L/CD27. Conversely, T cells from mice injected with AAV1 vector coding for the P2X7-potentiating 14D5-dimHLE construct showed enhanced sensitivity in this assay, notably at the lowest ATP dose of 30 µM (**Figures 1C–E**). Hence, these results confirm the blocking effect of 13A7-hcAb, and conversely the potentiating effect of 14D5-dimHLE, which were produced *in vivo* upon a single i.m. injection of the corresponding AAVnano vectors.

### Nanobody-based biologic that inhibit P2X7 functions have a beneficial anti-tumor effect in P2X7-expressing tumor models

**Figure 2:**
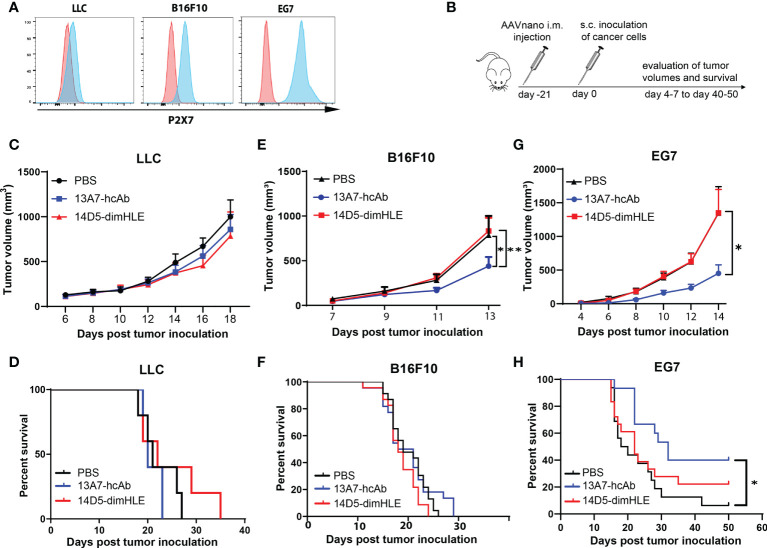
Antagonistic nanobody-based biologics directed against P2X7 inhibit the subcutaneous growth of tumor cell lines expressing P2X7 *in vivo* **(A)** P2X7 cell surface levels of LLC carcinoma, B16F10 melanoma, and EG7 thymoma cell lines were evaluated by flow cytometry using a fluorochrome-conjugated P2X7-specific antibody (blue histograms) and compared to the background staining obtained using a fluorochrome-coupled isotype control antibody (red histograms). **(B)** Protocol used to evaluate the *in vivo* effect of P2X7-blocking or P2X7-potentiating biologics on tumor growth. Mice were injected i.m. with 10^11^ vg/mouse of the indicated AAVnano vectors, namely AAV-13A7-hcAb (coding for the P2X7-blocking nanobody-based biologic), or AAV-14D5-dimHLE (coding for the P2X7-potentiating nanobody-based biologic). 21 days later, mice were inoculated with the indicated cancer cells in the right flank of the animal and tumor volumes were monitored over time. **(C, E, G)** Mean tumor volumes in groups of mice (n = 10) injected with the LLC carcinoma, B16F10 melanoma, or EG7 thymoma tumor model. **(D, F, H)** Corresponding survival curves are shown for each tumor model. Experiments were repeated at least 2 times with similar results. The statistical comparisons between tumor volumes were performed using two-way ANOVA. The statistical analyses of survival rates were performed using Log-Rank tests. *p<0.05, **p<0.01.

To evaluate the effects of the blocking and the potentiating nanobody-based biologics in a tumor context, we took advantage of our AAVnano methodology to induce their continuous expression *in vivo*. However, as expression of P2X7 in tumor cells has been reported to influence tumor growth *per se*, owing to a trophic effect of P2X7 tonic stimulation, we first verified the levels of P2X7 expression at the surface of each tumor model. The results demonstrated that Lewis lung carcinoma (LLC) cells express very low surface levels of P2X7, while the melanoma B16F10 cells display intermediate P2X7 levels, and the EG7 thymoma cells express the highest levels (**Figure 2A**). These tumor cells were inoculated in groups of animals that received AAV1 vectors coding for either 13A7-hcAb or 14D5-dimHLE three weeks before and the tumor volumes were measured on the following days (**Figure 2B**). For the LLC model with low levels of P2X7, we observed no significant effect of the nanobody-based biologics on tumor growth, but a tendency of better tumor control in the group that received the AAV vector coding for 14D5-dimHLE (**Figure 2C**), also resulting in a slightly better survival in this group (**Figure 2D**). In the B16F10 melanoma model, the potentiating 14D5-dimHLE biologic had no obvious effect on tumor growth and mice survival, while the antagonistic 13A7-hcAb significantly reduced tumor growth (**Figure 2E**) and slightly improved survival, although not significantly (**Figure 2F**). In the EG7 thymoma model with the highest level of P2X7, we observed again that the P2X7-potentiating biologic had no effect on the early phase of tumor growth. However, as also observed in the melanoma model, blocking P2X7 with the 13A7-hcAb biologic significantly decreased tumor growth and improved mice survival (**Figures 2G, H**). Interestingly, a gradation of the beneficial effect of the blocking 13A7-hcAb biologic can be noticed, apparently in relation with the surface expression of P2X7 in each of these tumor models (**Figures 2A, C, E, G**). Taken together, these results suggest that inhibition of P2X7 significantly reduces tumor growth when P2X7 is expressed at the surface of the tumor cells.

### Potentiation of P2X7 functions *in vivo* with the 14D5-dimHLE biologic influences the composition of immune infiltrates in a melanoma model

**Figure 3:**
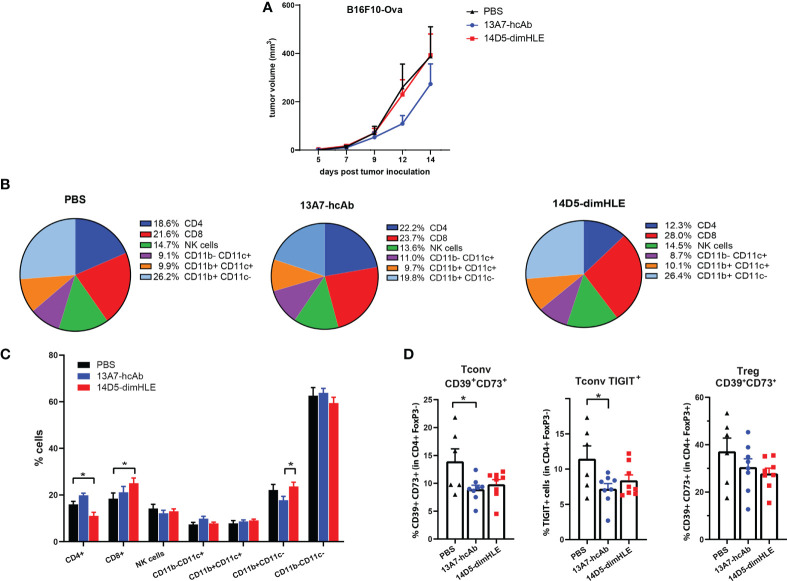
Modification of immune cell infiltrates within the tumor microenvironment in response to *in vivo* generated biologics targeting P2X7. **(A)** Tumor growth curves of B16F10-Ova melanoma cells are shown for group of mice treated as in **Figure 2B**. **(B, C)** Relative percentages of tumor infiltrating myeloid and lymphoid CD45^+^ cells were determined by flow cytometry 14 days after tumor inoculation in groups of mice (n=6-7) treated with AAV-13A7-hcAb, AAV-14D5-dimHLE, or with PBS. **(D)** Percentages of CD39^+^CD73^+^ cells (left panel) or TIGIT^+^ (middle panel) cells within the conventional CD4^+^FoxP3^-^ T cells (Tconv), and the percentages of CD39^+^CD73^+^ cells within the CD4^+^FoxP3^+^ Tregs subset (right panel) were determined by flow cytometry. The statistical comparisons between tumor volumes were performed using 2-way ANOVA. The statistical comparisons between tumor infiltrating cell subpopulations were performed using one-way ANOVA. *p<0.05.

Next, we turned to the B16F10-Ova melanoma model with the aim to also study the composition of immune infiltrates, and possibly to detect antigen-specific anti-tumor T cells that could emerge if an adaptive immune response is induced by nanobody-based therapies. As before, groups of mice received AAVnano vectors coding for 14D5-dimHLE or 13A7-hcAb. The monitored tumor growth was in line with the previous results obtained with the B16F10 model and showed again that 13A7-hcAb inhibits tumor growth in the early phase of tumor progression, although not significantly, while 14D5-dimHLE did not induce any conspicuous effect (**Figure 3A**). Next, 14 days after tumor inoculation, the tumor was resected to study the composition of the immune infiltrates in the different groups. We observed that the nanobody-based biologic 14D5-dimHLE alter the composition of the lymphoid compartment in the TME significantly as compared to control mice (**Figure 3B**). Indeed, 14D5-dimHLE biologic decreased the proportion of CD4^+^ T cells and increased the proportion of CD8^+^ T cells (**Figure 3C**). 13A7-hcAb biologic in contrast did not significantly modify the lymphoid compartment but reduced the proportion of CD11b^+^ CD11c^-^ myeloid cells (**Figures 3B, C**). Both 13A7-hcAB and 14D5-dimHLE biologics decreased the percentage of CD4^+^ T cells expressing the exhaustion marker TIGIT, and more importantly, of those expressing the immunosuppressive enzymes CD39 and CD73, two important purinergic checkpoints (**Figure 3D**). These ecto-enzymes tended also to be less expressed at the surface of Tregs, although with a higher variability. Although the composition of the TME was modified by the biologics treatment, with a reduction of immunosuppressive cells This was apparently not enough to reject the tumor in agreement with the absence of detectable CD8^+^ T cells recognizing the tumor-derived ovalbumin (Ova) antigen (data not shown). These results indicate that both P2X7-targeting biologics can influence the composition of the TME, even the potentiating 14D5-dimHLE biologic that did not show any effect on the tumor growth in this model. Taken together, this suggests that although P2X7 potentiation induces a more favorable TME for the emergence and for the activation of anti-tumor immune cells, targeting P2X7 alone was clearly not enough to control tumor progression in this aggressive melanoma model.

### P2X7 potentiating and blocking biologics in combination with an immunogenic chemotherapy improve tumor control in the EG7 tumor model

**Figure 4:**
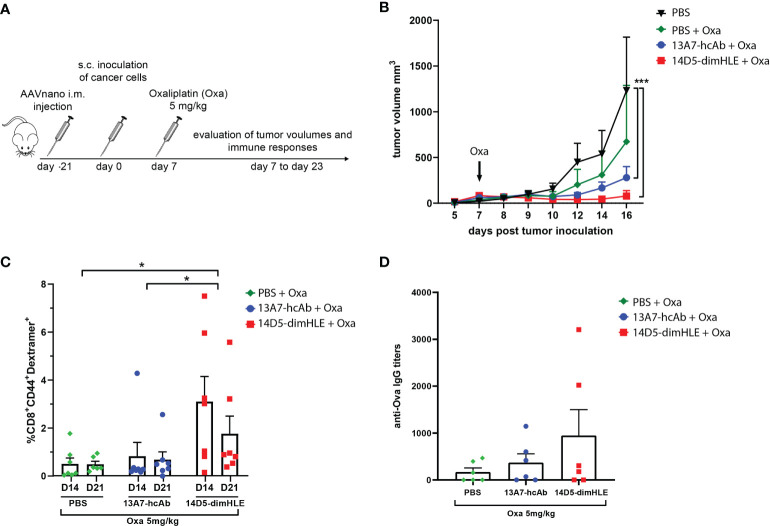
Treatment with P2X7-targeting biologics combined with oxaliplatin chemotherapy **(A)** Protocol used to evaluate the *in vivo* effect of P2X7-targeting biologics on tumor growth when combined with oxaliplatin chemotherapy. AAVnano coding for the P2X7-blocking 13A7-hcAb, or for the P2X7-potentiating 14D5-dimHLE were injected i.m. 21 days before s.c. injection of EG7-Ova thymoma cells. Oxaliplatin (5 mg/kg) was injected once at day 7 post tumor inoculation. **(B)** Mean tumor volumes overtime in each group of animals (n=7). **(C)** Percentages of Ova-specific CD8^+^ cells in peripheral blood were determined by flow cytometry at days 14 and 22 post tumor inoculation. **(D)** Anti-Ova IgG titers were determined by ELISA in sera collected 23 days post tumor inoculation. Statistical comparisons between tumor volumes were performed using two-way ANOVA, and statistical comparisons between the levels of anti-Ova immune responses were performed using one-way ANOVA *p<0.05, ***p<0.001.

We next evaluated our P2X7-targeting biologics in combination with an immunogenic chemotherapy treatment. We reasoned that such a treatment should indeed increase the concentration of ATP in the TME and may therefore synergize with P2X7 targeting. We chose the EG7 model, as it was previously shown to be sensitive to oxaliplatin and to induce an immunogenic cell death that relies on ATP release *in vivo* (28). Notably, we expected that the potentiating 14D5-dimHLE nanobody-based biologic would favor the emergence of anti-tumor immune responses and promote tumor control when given in combination with oxaliplatin. To evaluate this hypothesis, we injected as before the AAVnano vectors coding for both biologics three weeks before EG7 tumor inoculation. A single dose of oxaliplatin was then injected 7 days after tumor inoculation when the mean volume of tumors reached 50 mm^3^ (**Figure 4A**). As expected, oxaliplatin used at 5 mg/kg decreased tumor growth but did not induce complete tumor rejection. When combined with the 14D5-dimHLE potentiating biologic, a better tumor control was observed and, importantly, tumor rejections in most mice (**Figure 4B**). Taking advantage of the expression of the Ova antigen by this tumor cell line, we also monitored the emergence of anti-Ova CD8^+^ specific T cell in the blood of these mice, 14 and 22 days post tumor inoculation. The data indeed suggest that the combination of oxaliplatin and 14D5-dimHLE results in more robust CD8^+^ T cells stimulation than in the other groups (**Figure 4C**). This was also the case when evaluating the anti-Ova IgG responses, suggesting a higher level of immune stimulation in this group (**Figure 4D** **)**. Interestingly, we also noticed a better tumor control in the group of mice that received oxaliplatin together with the P2X7-blocking 13A7-hcAb biologic (**Figure 4B**). As the EG7 tumor cell line expresses high levels of P2X7, we assume that this effect was related to the inhibition of the tonic stimulation of P2X7. This is in agreement with the lower stimulation of immune cells in this group in comparison with the previous one (**Figures 4C, D**). Taken together, these data suggest that both P2X7-targeting biologics, the blocking 13A7-hcAbs as well as the potentiating 14D5-dimHLE, can improve tumor control in combination with oxaliplatin chemotherapy, although with different mechanisms of action.

### A bispecific biologic targeting both CD73 and PD-L1 more effectively inhibits tumor growth *in vivo* than monotherapies with P2X7-specific biologics

**Figure 5:**
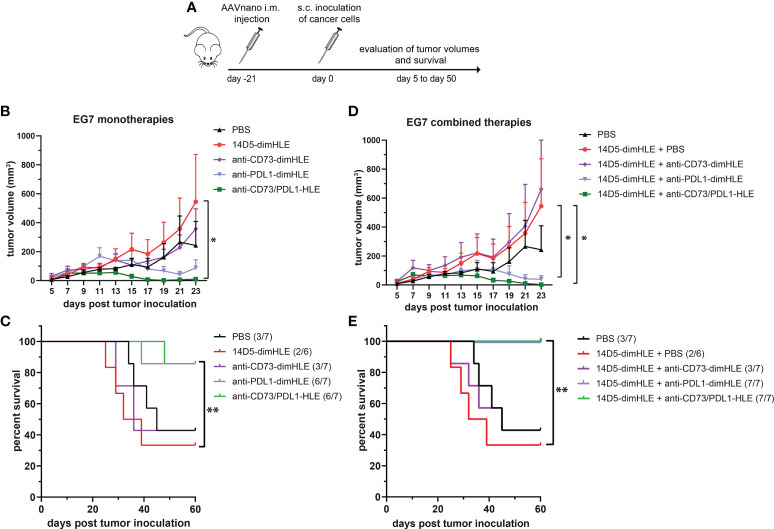
A bispecific biologic targeting both CD73 and PD-L1 more effectively inhibits the subcutaneous growth of EG7 melanoma cells *in vivo* than monotherapies with P2X7-specific biologics. **(A)** Protocol used to evaluate the *in vivo* effect of nanobody-based biologics targeting P2X7, CD73, and/or PD-L1. The AAVnano coding for the P2X7-potentiating 14D5-dimHLE, for the CD73-inhibiting anti-CD73-dimHLE, for the PD-L1 blocking anti-PDL1-dimHLE, or the bispecific biologic targeting both CD73 and PD-L1 (anti-CD73/PDL1-HLE), were injected i.m. 21 days before s.c. injection of EG7 thymoma cells. These biologics were either evaluated alone **(B, C)**, or in combination with the 14D5-dimHLE P2X7-potentiating biologic **(D, E)**. In that case, the AAVnano vectors coding for 14D5-dimHLE were injected i.m. into the gastrocnemius while the AAVnano vectors coding for the additional biologics were injected i.m. into the quadriceps. Mean tumor volumes overtime **(B, D)** and survival **(C, E)**, were followed in the different group of mice (n=7), as indicated. The statistical comparisons between tumor volumes were performed using two-way ANOVA, and the statistical analyses of survival were performed using Log-Rank tests. *p<0.05, **p<0.01.

**Figure 6:**
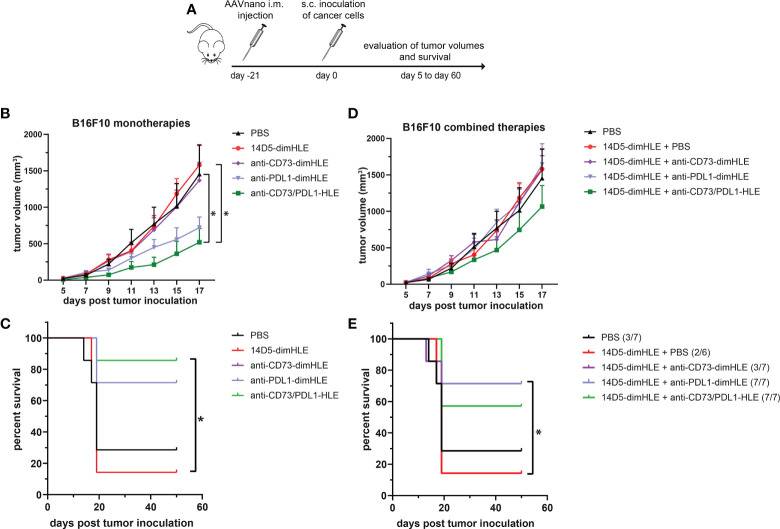
A bispecific biologic targeting both CD73 and PD-L1 more effectively inhibits the subcutaneous growth of B16F10 melanoma cells *in vivo* than monotherapies with P2X7-specific biologics **(A)** Protocol used to evaluate the *in vivo* effect of nanobody-based biologics targeting P2X7, CD73, and/or PD-L1. The AAVnano coding for the P2X7-potentiating 14D5-dimHLE, for the CD73-inhibiting CD73-dimHLE, for the PD-L1 blocking PDL1-dimHLE, or the bispecific CD73/PDL1-HLE, were injected i.m. 21 days before s.c. injection of B16F10 melanoma cells. These biologics were either evaluated alone **(B, C)**, or in combination with the 14D5-dimHLE P2X7-potentiating biologics **(D, E)**. In that case, the AAVnano vectors coding for 14D5-dimHLE were injected i.m. into the gastrocnemius while the AAVnano vectors coding for the additional biologics were injected i.m. into quadriceps. Mean tumor volumes overtime **(B, D)** and survival **(C, E)**, were followed in the different group of mice (n=7), as indicated. The statistical comparisons between tumor volumes were performed using two-way ANOVA, and the statistical analyses of survival were performed using Log-Rank tests. *p<0.05.

We next evaluated other nanobody-based biologics using our method, either alone or in combination with 14D5-dimHLE treatment. As CD73 has emerged as a key purinergic checkpoint in the TME, we designed a CD73-specific construct based on nanobody clone SB121, which displays an antagonistic effect on the enzymatic activity of CD73 *in vitro*. Like 14D5-dimHLE, the CD73-targeting construct, termed anti-CD73-dimHLE, contains a nanobody dimer (SB121), coupled to an anti-albumin nanobody to increase its half-life *in vivo*. For comparison, we also designed a nanobody-based construct that targets the well-known PD-L1 immune checkpoint, that we termed anti-PDL1-dimHLE. For that, we used the same dimHLE format but with nanobody clone B3, previously demonstrated to specifically block the interaction between PD-L1 and its cognate receptor PD-1 (46). Finally, we also designed a bispecific construct targeting both, CD73 and PD-L1, as both proteins are overexpressed by the tumor cells and/or by the immune infiltrates that compose the TME (54). We reasoned that this bispecific construct, termed anti-CD73/PDL1-HLE, might not only improve T cell activation by inhibiting the PD-1/PD-L1 axis, but also inhibit the accumulation of anti-inflammatory adenosine in the TME, which represents another non-redundant immunosuppressive mechanism. Evaluation of the monospecific and the bispecific nanobody-based biologics was again performed using our AAVnano method using the EG7 thymoma model (**Figure 5**) as well as the more aggressive and less immunogenic B16F10 melanoma model (**Figure 6**). For that, AAV8 vectors coding for either anti-CD73-dimHLE, anti-PDL1-dimHLE or anti-CD73/PDL1-HLE were generated and injected i.m. in different groups of mice. For comparison, an additional group was injected with the AAVnano vector coding for 14D5-dimHLE, the P2X7-potentiating biologic. As before, EG7 or B16F10 tumor cell lines were inoculated 3 weeks later (**Figures 5A** and **6A**). Like in the previous experiments, the 14D5-dimHLE biologic alone did not promote tumor control of EG7 nor of B16F10 tumors, and was statistically indistinguishable from the untreated control group, both in terms of tumor growth and survival (**Figures 5B, C** and **6B, C**). Similarly, the anti-CD73-dimHLE biologic did not inhibited tumor growth nor survival in both tumor models. In contrast, the bispecific anti-CD73/PDL1-HLE biologic significantly enhanced the control of tumor growth in the B16F10 melanoma model, or even led to complete tumor rejection in the EG7 model, as well as improved mice survival in both tumor models (**Figures 5B, C** and **6B, C**). Interestingly, in both tumor models, this novel bispecific construct also tended to be more efficient than the anti-PDL1-dimHLE biologic, in terms of reduction of tumor growth as well as in terms of mice survival.

Finally, we also evaluated each of the three biologics (anti-CD73-dimHLE, anti-PDL1-dimHLE, and anti-CD73/PDL1-HLE), in combination with the 14D5-dimHLE. For that, an AAV1 vector coding for 14D5-dimHLE was injected in the gastrocnemius while the AAV8 vector coding for the second biologic was injected in the quadriceps to induce concomitant expression of two biologics at the same time. As in the previous experiment, EG7 or B16F10 tumor cell lines were inoculated 3 weeks later (**Figures 5A** and **6A**). In both tumor models, the anti-CD73-dimHLE did not significantly synergize with the 14D5-dimHLE biologic, neither on the control of tumor growth nor in terms of mice survival (**Figures 5D, E** and **6D, E**). In contrast, tumor growth was better controlled and mice survival was significantly improved in the groups that received anti-PDL1-dimHLE, or anti-CD73/PDL1-HLE, in addition to the 14D5-dimHLE biologic, suggesting at least that the beneficial effects of anti-PDL1-dimHLE, and of anti-CD73/PDL1-HLE are maintained in the presence of the P2X7-potentiating biologic. However, as compared to the data obtained for each individual construct, the results did not show a better anti-tumor effect in combination with 14D5-dimHLE biologic. In fact, this was difficult to evaluate in the less aggressive EG7 tumor model as both mono and combined therapy led to near complete tumor rejection and survival of almost all the mice in these cohorts (**Figures 5B vs D, C vs E**). However, when comparing the data in the more aggressive B16F10 melanoma model, the combined therapy tended to be slightly less efficient than anti-PDL1-dimHLE or anti-CD73/PDL1-HLE given alone (**Figures 6B vs D, C vs E**). This would suggest that, by favoring the stimulation of P2X7 on the surface of the tumor cells, the potentiating 14D5-dimHLE in the long run displays a net effect that is rather in favor of tumor progression than in favor of the anti-tumor immune response. This may suggest that short term P2X7 potentiation, by means of direct injection of the recombinant 14D5-dimHLE biologic, may be better suited than its continuous stimulation, to tip the balance in favor of the activation of the immune system. However, this would have to be tested in future experiments in a broader range of tumor types that express, or not, P2X7. Nevertheless, from the present data, we concluded that targeting both, CD73 and PD-L1, with bispecific nanobody-based biologics represent a promising approach in cancer treatment.

## Discussion

The TME is a dynamic environment and the privileged site where cancer cells are in close contact with the host. The biochemical and cellular composition of the TME is of paramount importance for the regulation of cancer cells metabolism, proliferation, motility and dissemination. The TME can facilitate anti-tumor immune responses but may conversely foster the generation of an immunosuppressive environment that facilitates tumor growth. The biochemical composition of the TME is a result of the activity of the cancer and host cells. Over the last few years, the abundance of eATP was identified as a prominent TME characteristic (29, 55). Cell stress and cell death lead to loss of plasma membrane integrity and represent important sources of eATP in the TME (11). Hypoxia itself is a potent stimulus for ATP release, even in the absence of cell damage. Various cells also release ATP, either as part of their normal metabolism or when responding to activation, metabolic or mechanical stress, or signals that induce cell death. This has been documented for numerous cells, including cancer cells, dendritic cells, tumor-infiltrating neutrophils, tumor-associated macrophages or platelets (29, 31, 32, 55). Once released in the TME, eATP binds to P2Y and P2X receptors. Among the P2 receptors, the role of the P2X7 receptor has been widely implicated in several types of cancer and suggested to be involved in the complex dialog between cancer and immune cells within the TME (31). The balance between ATP release and its degradation by a variety of ecto-enzymes determines the concentration of eATP and of its catabolites in the TME. eATP is indeed catabolized to ADP and AMP, and then to the immunosuppressive adenosine that exerts its effect *via* the widely expressed P1 receptors. Generation of adenosine from eATP is essentially controlled by the sequential activity of CD39 and CD73. These ecto-enzymes are expressed by many cell types in the TME, including cancer cells, cancer-associated fibroblasts (CAFs), cytotoxic T cells, Tregs, NK cell subsets, M2-like tumor associated macrophages (TAMs), or myeloid-derived suppressor cells (MDSCs) (29). Accumulation of adenosine in the TME exert a potent immunosuppressive function, notably through the inhibitory A2a adenosine receptors expressed by T cells that restraint their activation and expansion in the adenosine riche TME (36–38). Preclinical and clinical studies have further highlight the potential offered by therapeutic strategies aiming to inhibit adenosine signaling in cancer, and the “purinergic signature” that favor their application (39–42).

In this study, we used the AAVnano method approach (43, 44) to evaluate the anti-tumor potential of several nanobody-based biologics in different tumor models. Nanobodies, as for classical antibodies, offer excellent target specificity that may prevent unwanted off-target effects possibly observed with small chemical drugs. We selected nanobodies that specifically bind mouse P2X7 and but not the close P2X4 and P2X1 paralogs (9, 45). We chose to express our nanobody-based biologics directly *in vivo* upon AAV vector-mediated gene transfer. AAV vectors are widely used in gene therapy settings and represent an efficient and safe approach to transfer genes of interest into muscle cells and to elicit long-term systemic *in situ* production of transgenic proteins, including selected antibodies that confer protection from infectious diseases (56, 57). In our experimental approach, we previously demonstrated that a single i.m. administration of the AAVnano vector (10^11^ vg/mouse) coding for our engineered nanobody-based biologics was sufficient to elicit *in vivo* production of saturating levels of biologics that persist for several months. We validated here, prior to the tumor inoculation, that the blocking or the potentiating P2X7-specific biologics were produced *in vivo* and were able to completely block P2X7 (i.e., for the 13A7-hcAb biologic) or to potentiate its activity notably at low ATP concentration, (i.e., for the 14D5-dimHLE construct) (**Figure 1**, and data not shown). As a hallmark associated with P2X7 receptor activation, we used a sensitive *ex vivo* assay based on metalloprotease-dependent shedding of CD27 and CD62L from the T cell surface (5, 20–23). Our data demonstrate that a single i.m. injection of AAVnano coding for the P2X7-antagonistic 13A7-hcAb protects T cells from ATP-induced CD27 and CD62L shedding (**Figures 1C–E**) while the AAVnano coding for the P2X7-potentiating 14D5-dimHLE sensitized T cells to ATP-induced shedding of CD27 and CD62L (**Figures 1C–E**). We next used our AAVnano methodological approach to evaluate the net consequence of either blocking, or potentiating, P2X7 in different tumor models. The tumor models were selected for their differential levels of P2X7 surface expression, ranging from very low, in the lung carcinoma model LLC, intermediate in the B16F10 melanoma model, to relatively high expression in the EG7 thymoma model (**Figure 2A** **)**. As P2X7 tonic stimulation in the context of the ATP-rich TME has been associated with tumor growth and invasiveness, it appeared indeed to be an important factor to consider in our study. Importantly, none of these cancer cell lines is rapidly going into cell death by concentrations of ATP below the millimolar range, while this is the case for mouse T cells (not shown). This indicates, as suggested by different studies, that the P2X7 receptor in cancer cells does not behave as a cytolytic receptor. This may be explained by the expression of particular splice variants, or mutated versions of the receptor, that remain to be characterized in our tumor lines. However, this was studied in a wide variety of human cancer cells, where P2X7 variants that have lost their cytolytic properties but have retained the capacity to trigger calcium influx in response to ATP (31, 58–62). Therefore, blocking P2X7 is expected to inhibit tonic tumor cells stimulation and tumor progression. Our results are in complete agreement with this hypothesis. We demonstrate that blocking P2X7 in the LLC lung carcinoma model had no effect on the tumor progression and mice survival, in line with the very low surface expression of P2X7 in this tumor line (**Figures 2A, C, D**). For the two other tumor models, our data point to a gradation of the beneficial effect of the 13A7-hcAb biologic, in direct relation with the surface expression of P2X7 in each of these tumor models. Indeed, the P2X7-blocking 13A7-hcAb biologic more potently inhibits tumor growth in the EG7 tumor model, which displays the highest P2X7 surface level, than in the B16F10 model that expresses an intermediate level of P2X7 (**Figures 2A, C, E, F**). Taken together, these results suggest that inhibition of P2X7 significantly reduces tumor growth and improves survival, when P2X7 is expressed at the surface of the tumor cells.

To better understand the biological effects of the nanobody-based biologics targeting P2X7 on the composition of the TME, we next studied the frequency and the phenotype of the cells present in the tumor infiltrates. For that, we resected the tumor and analyzed the cell profiles by flow cytometry. We observed that the nanobody-based biologic 14D5-dimHLE, that was paradoxically not associated with any beneficial effect on tumor growth nor on mice survival, significantly alters the composition of the lymphoid compartment in the TME as compared to control mice (**Figures 3B, C**). Notably, the 14D5-dimHLE biologic was found to increase the proportion of CD8^+^ T cells, which may indicate the stimulation of tumor-specific T cells (**Figure 3C**). This treatment was also associated with a decrease in the expression of the exhaustion marker TIGIT, and of CD39 and CD73, two ecto-enzymes considered as targetable purinergic checkpoints (32–35). This suggests that P2X7 potentiation by the 14D5-dimHLE biologic contributes to the induction of a more favorable TME, which possibly is less immunosuppressive, but that this treatment alone is not sufficient for the emergence of a potent and effective anti-tumor immune response. As this result has been obtained in the B16F10 melanoma model expressing intermediate levels of P2X7, higher tonic stimulation of P2X7 at the surface of the tumor cells, facilitated by the 14D5-dimHLE biologic, probably also contributed to the global effect of this biologic *in vivo*. This may be addressed in future studies using for instance a B16F10 melanoma model deficient for P2X7 expression, to better delineate the potential beneficial effect of P2X7 stimulation on immune cells, from its detrimental effect on the tumor cells themselves.

As the 14D5-dimHLE biologic does not promote beneficial effects when used alone, we next evaluated whether potentiation of P2X7 can enhance anti-tumor immune responses when combined with immunogenic oxaliplatin chemotherapy, that may increase the release of ATP in the tumor, or with anti-immune checkpoints therapy, or antagonism of CD73 involved in the formation of the immunosuppressive adenosine. We first evaluated the combination therapy based on a single injection of oxaliplatin in animals that have been injected beforehand with AAVnano vectors coding for the 14D5-dimHLE biologic (**Figure 4A**). We observed that this combination resulted in a significant decrease in EG7 tumor growth (**Figure 4B**), associated with an increase in tumor-specific cellular and humoral immune responses (**Figures 4C–D**). These data suggest that P2X7 potentiation in the context of an immunogenic chemotherapy reinforce the immune responses. The mechanism may involve stimulation and migration of dendritic cells, as well as Treg cell death, which both involve P2X7 and can contribute together to the emergence of effective adaptive anti-tumor immune responses (4, 26, 28, 29).

We then evaluated the 14D5-dimHLE P2X7-potentiating biologic in combination with a nanobody-based biologics targeting CD73, another important purinergic checkpoint, or PD-L1, a prototypic immune checkpoint. Another construct was designed to target both CD73 and PD-L1 at the same time using a bispecific nanobody-based biologic. We evaluated theses biologics as monotherapy and in combination with 14D5-dimHLE in the EG7 thymoma model and in the less immunogenic B16F10 melanoma model considered to be more aggressive. We did not observe any beneficial effect of these treatments when combined with 14D5-dimHLE, as compared to their evaluation as monotherapy (**Figures 5** and **6**). In fact, it was not possible to faithfully evaluate the possible beneficial effect of the combination involving anti-PDL1-dimHLE or anti-CD73/PDL1-HLE biologics as both treatments were already very effective alone to induce tumor control. Nevertheless, the treatment based on anti-CD73-dimHLE and 14D5-dimHLE, either as monotherapy, or combined, was not effective to control tumor growth nor to improve mice survival. This may at least partly reflect the P2X7-dependant trophic stimulation of EG7 and B16F10 cancer cells. Blocking CD73 may indeed increase the accumulation of ATP in the TME and 14D5-dimHLE may further enhance P2X7 activity at the surface of these cancer cells, tipping the balance in favor of tumor growth. Whether this is a plausible explanation may deserve further investigations, involving for instance the assessment of eATP concentration in the TME and its evolution upon treatment with these biologics.

In the course of our evaluations, we tested for the first time a bispecific biologic targeting CD73 and PD-L1, that we termed anti-CD73/PDL1-HLE. Here, we aimed to combine a nanobody targeting the prototypical PD-L1 immune checkpoint with a nanobody targeting CD73 that is considered as an emerging targetable purinergic checkpoint. This anti-CD73/PDL1-HLE biologic improved the control of tumor growth in the aggressive B16F10 melanoma model, or even induced complete tumor rejection in the EG7 model, as well as improved mice survival in both tumor models (**Figures 5B, C** and **6B, C**). Interestingly, in both tumor models, this novel bispecific biologic also tended to be more efficient than the anti-PDL1-dimHLE biologic, in terms of reduction of tumor growth as well as in terms of mice survival. The mechanism remains to be fully explored, but possibly rely on the inhibition of two complementary non-redundant mechanisms that contribute together to the progressive formation of the immunosuppressive TME. In line, previous studies demonstrated that antibodies directed against CD73 enhance antitumor immune responses, when used in combination with anti-immune checkpoints (34, 35, 63). Also, as both CD73 and PD-L1 are overexpressed in the TME, this bispecific biologic may advantageously accumulate around the tumor to exert its beneficial effect but this remains to be fully investigated. We conclude, however, from our present data that targeting both, CD73 and PD-L1, with bispecific nanobody-based biologics represents a promising approach in cancer treatment.

## Data availability statement

The raw data supporting the conclusions of this article will be made available by the authors, without undue reservation.

## Ethics statement

The animal study was reviewed and approved by French Ministry of Education and Research, after consultation of the ethical committee.

## Author contributions

MD, FK-N, and SA conceived and design the study. MD, AS, RH, HG, CA, MB, SM, YD, BR, TM, AM, FK-N and SA developed the methodology. All authors contributed to the planning, acquisition of data, or to the interpretation of the results. MD, CA, FK-N, and SA wrote the manuscript. All authors revised and approved the manuscript.

## Funding

The present work was funded by a grant No ANR-18-CE92-0046-01 from Agence National de la Recherche (ANR) to SA, and by grants No 310/13, SFB1328-A13, SFB1328-Z02 from the Deutsche Forschungsgemeinschaft (DFG) to FK-N, BR, and TM.

## Acknowledgments

The authors would like to thank Rachid Zoubairi, Gaetan Riou, Laetitia Jean and Chantal Barou, from Rouen, and Birte Albrecht and Dorte Wendt, from Hamburg, for excellent technical assistance.

## Conflict of interest

The authors declare that the research was conducted in the absence of any commercial or financial relationships that could be construed as a potential conflict of interest.

FK-N is a co-inventor on a patent application on P2X7-specific nanobodies. FK-N, SM, YD, BR, and TM are co-inventors on a patent application on CD73-specific nanobodies.

## Publisher’s note

All claims expressed in this article are solely those of the authors and do not necessarily represent those of their affiliated organizations, or those of the publisher, the editors and the reviewers. Any product that may be evaluated in this article, or claim that may be made by its manufacturer, is not guaranteed or endorsed by the publisher.
